# How Machine Learning and Statistical Models Advance Molecular Diagnostics of Rare Disorders Via Analysis of RNA Sequencing Data

**DOI:** 10.3389/fmolb.2021.647277

**Published:** 2021-06-01

**Authors:** Lea D. Schlieben, Holger Prokisch, Vicente A. Yépez

**Affiliations:** ^1^ School of Medicine, Institute of Human Genetics, Technical University of Munich, Munich, Germany; ^2^ Institute of Neurogenomics, Helmholtz Zentrum München, Neuherberg, Germany; ^3^ Department of Informatics, Technical University of Munich, Munich, Germany

**Keywords:** rare disorders, RNA sequencing, machine learning, statistical models, aberrant expression, aberrant splicing, mono-allelic expression

## Abstract

Rare diseases, although individually rare, collectively affect approximately 350 million people worldwide. Currently, nearly 6,000 distinct rare disorders with a known molecular basis have been described, yet establishing a specific diagnosis based on the clinical phenotype is challenging. Increasing integration of whole exome sequencing into routine diagnostics of rare diseases is improving diagnostic rates. Nevertheless, about half of the patients do not receive a genetic diagnosis due to the challenges of variant detection and interpretation. During the last years, RNA sequencing is increasingly used as a complementary diagnostic tool providing functional data. Initially, arbitrary thresholds have been applied to call aberrant expression, aberrant splicing, and mono-allelic expression. With the application of RNA sequencing to search for the molecular diagnosis, the implementation of robust statistical models on normalized read counts allowed for the detection of significant outliers corrected for multiple testing. More recently, machine learning methods have been developed to improve the normalization of RNA sequencing read count data by taking confounders into account. Together the methods have increased the power and sensitivity of detection and interpretation of pathogenic variants, leading to diagnostic rates of 10–35% in rare diseases. In this review, we provide an overview of the methods used for RNA sequencing and illustrate how these can improve the diagnostic yield of rare diseases.

## Introduction

Rare diseases are defined as life-threatening or chronically debilitating diseases with a low prevalence (<5 in 10,000) (Moliner and Waligora, 2017). Between 263 and 446 million individuals are currently affected worldwide (Nguengang Wakap et al., 2020). The majority, ∼80%, of rare disorders are of genetic origin. Rare genetic disorders are predominantly caused by rare variants in a single gene (Pogue et al., 2018). Identification of causal variants confirms the clinical diagnosis in patients with a suspected disorder, further allowing for suitable treatment options, early interventions, and genetic counseling of family members. The advent of next-generation sequencing technologies about a decade ago has transformed the diagnostic workflow by streamlining thousands of diagnostic assays into just a few. Rapidly decreasing costs, automation of high-throughput sequencing technologies, and advances in bioinformatic approaches facilitated the implementation of genome sequencing into routine diagnostics and a diagnosis can—in principle—now be made for nearly every patient with a genetic disorder (Sawyer et al., 2016; Stenton et al., 2020). Nevertheless, this is still not reached and the causative variant or associated gene cannot be determined in many patients by DNA sequencing due to limited knowledge about genotype–phenotype associations of rare variants.

By focusing on the ∼2% coding regions of the human genome, the molecular diagnostic rate of whole exome sequencing (WES) for the detection of causal pathogenic variants in children with suspected genetic disease is about 35% (Clark et al., 2018). Extending the genetic analyses to the noncoding regions increases the diagnostic yield to 41% by whole genome sequencing (WGS) (Clark et al., 2018). As a result, the majority of patients with rare genetic disorders do not receive a genetic diagnosis and remain unsolved.

Assuming a high penetrance of genetic variants in Mendelian disorders, rare genetic disorders have to be caused by rare variants. With 40,000–200,000 rare variants (minor allele frequency <0.005) present in a typical human genome (The 1000 Genomes Project Consortium, 2015), they are largely abundant. However, many are private, and only for a few, the functional consequences are known, restricting prioritization of potentially pathogenic variants (Uricchio et al., 2016). Guidelines for the interpretation of sequence variants have been developed by the American College of Medical Genetics and Genomics and the Association for Molecular Pathology [ACMG/AMP; (Richards et al., 2015)], integrating population, computational, functional, and segregation data. A variety of computational tools to predict the deleteriousness of a variant [CADD, MetaLR; (Dong et al., 2014; Kircher et al., 2014)], and the impact of a variant on protein function [PolyPhen-2, SIFT, and MutationAssessor; (Ng and Henikoff, 2001; Adzhubei et al., 2010; Reva et al., 2011)] and on splicing [spliceAI and MMsplice; (Cheng et al., 2019; Jaganathan et al., 2019)] has been developed. Functional validation can further confirm or disprove these predictions. Specifically, profiling the transcriptional level of a tissue at a defined time using RNA sequencing (RNAseq) can help to identify and prioritize pathogenic variants in three situations: 1) altered expression levels, 2) abnormal splicing events, and 3) detection of mono-allelic expression.

In 2016, the first studies systematically using RNAseq from muscle biopsies and fibroblasts cell lines to increase the diagnostic rate of rare disorders were released. They used arbitrary thresholds (Cummings et al., 2016, 2017) and statistical methods (Kremer et al., 2016, 2017) to call transcript aberrations. Since then, the bioinformatic approaches applied to RNAseq data have been advanced with machine learning methods such as OUTRIDER or FRASER, translating into more precise outlier calling (Brechtmann et al., 2018; Mertes et al., 2021). Machine learning methods are known to provide more robust predictions than statistical models (Bzdok et al., 2018). The diagnostic potential of complementing DNA sequencing with RNAseq for solving previously inconclusive WES cases is unequivocal. RNAseq-based diagnostics has led to a diagnostic yield of 7.5–18% in rare disease cohorts with no prior patient restrictions (Kremer et al., 2017; Frésard et al., 2019; Murdock et al., 2021; Yépez et al., 2021b) and of 35% in a cohort of patients including cases with predicted splice defects (Cummings et al., 2017; Gonorazky et al., 2019). In these studies, RNAseq led to the validation of novel pathogenic variants in known disease genes [e.g., chr21:47,409,881 C>T in the COL6A1 gene in Cummings et al. (2017)], but also to the discovery and validation of a new disease gene, TIMMDC1, where a deep intronic variant caused the activation of a cryptic splice site in two unrelated families (Kremer et al., 2017). RNAseq has been performed in the clinically accessible tissues whole blood, skeletal muscle, and skin-derived fibroblasts.

**FIGURE 1:**
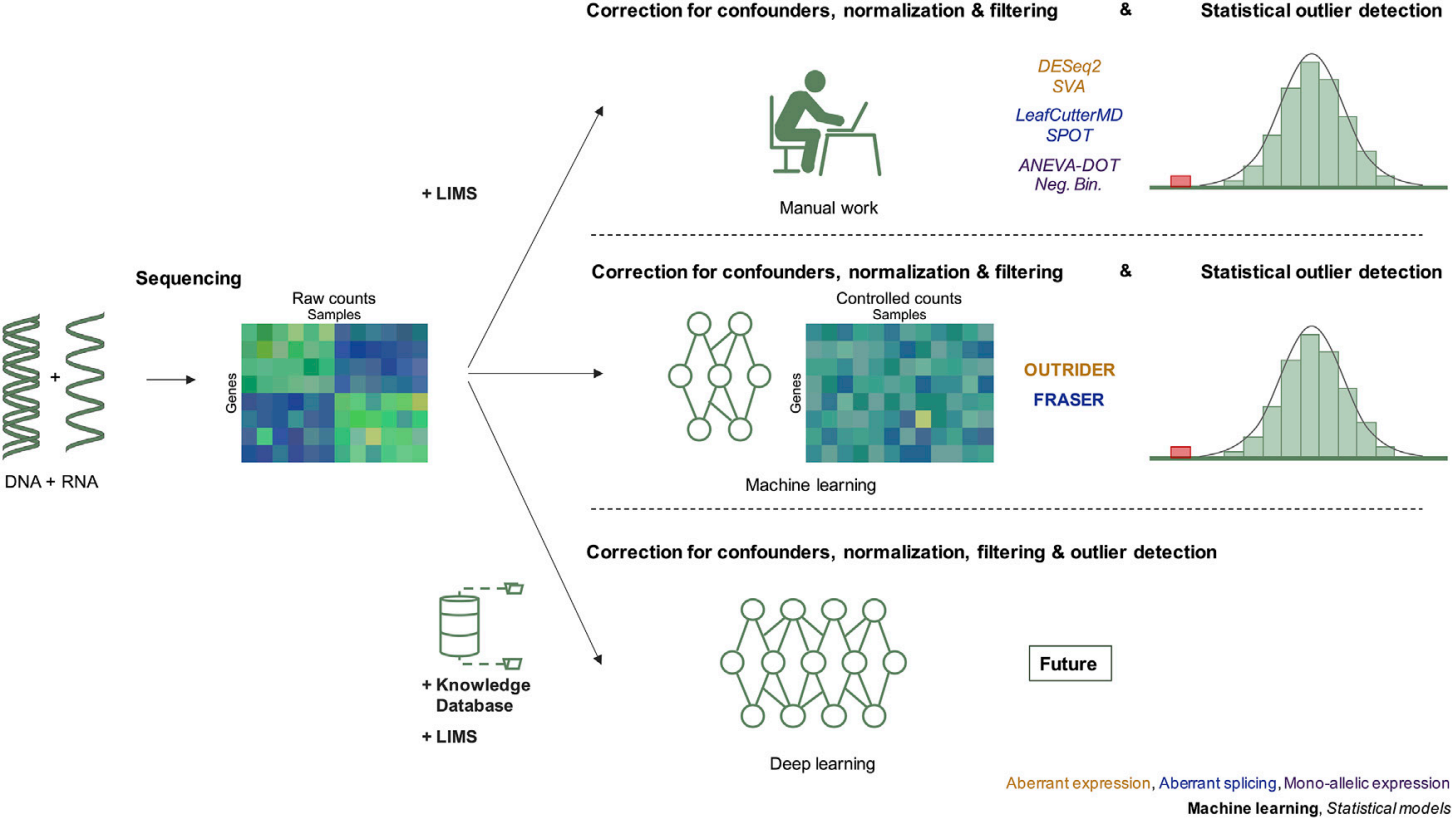
Evolution of RNAseq data modeling in the diagnosis of rare genetic disorders. The integration of RNAseq in molecular diagnostics includes three steps, data generation by DNA and RNA sequencing resulting in raw counts, correction for confounders, and detection of outliers after statistical modeling. Before 2018, the correction for confounders was based on manual work considering known technical and biological variables recorded in the LIMS. Since 2018, methods applying machine learning have been introduced. We predict that in the future, deep learning approaches will integrate knowledge databases, sequencing data and combine normalization, and statistical outlier detection. Next to each diagram, the different methods used to detect RNAseq outliers of the corresponding cases are given. LIMS, laboratory information management system.

The implementation of multi-omics data in rare genetic disorders is calling for new methods of machine learning and statistical algorithms to remove sample covariation and detect expression or splicing outliers (Figure 1). In this review, we provide an overview of the methods used for the detection of aberrant expression, aberrant splicing, and mono-allelic expression in the context of rare disease diagnostics. In addition, we illustrate how these can improve the diagnostic yield of rare diseases.

## Aberrant Expression

Previous studies demonstrated the ability of genetic variation to influence gene expression (Montgomery et al., 2010; Hulse and Cai, 2013; Li et al., 2014; Zhao et al., 2016). Gene expression levels outside the physiological range, the so-called gene expression outliers, are associated with Mendelian and common disorders. In common disorders, there is the concept that the combination of many variants contributes to the disease risk. Those genetic risk factors also include variants causing aberrant expression and can be summarized in polygenic risk scores. On the other hand, Mendelian disorders are monogenic. Despite most Mendelian disorders being caused by variants in the protein-coding region of the DNA, aberrant expression events are, however, frequently caused by noncoding variants in regions such as enhancers, promoters, and suppressors, as well as by RNA degradation via nonsense-mediated decay (NMD) (Zeng et al., 2015; Li et al., 2017). Although gene expression altering variants can be detected by DNA sequencing, the functional consequences are difficult to predict. The quantification of RNA abundance and primary structure by RNAseq allow them to directly measure the consequences of genetic variants on gene expression.

**FIGURE 2:**
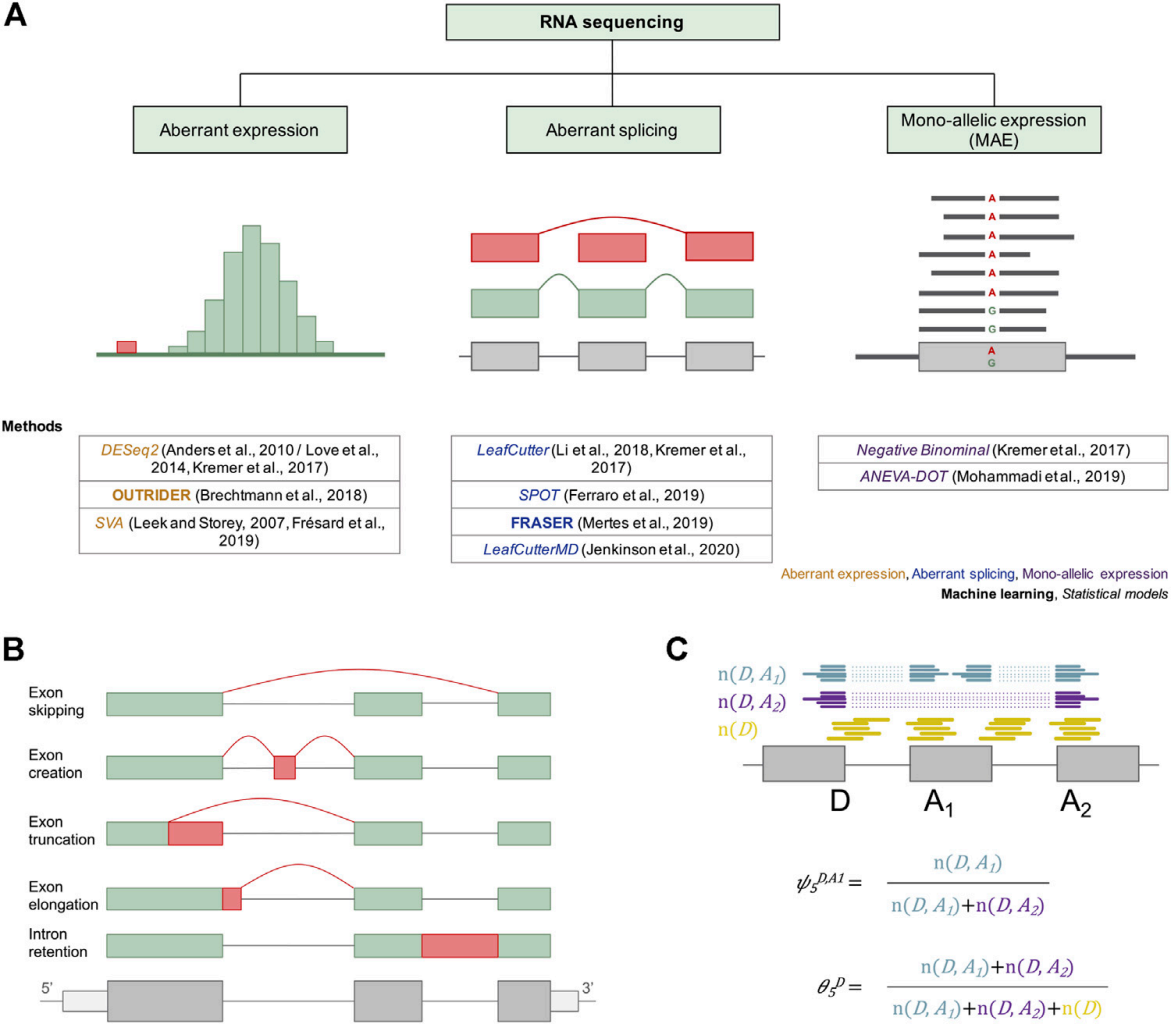
Current approaches for the molecular diagnosis of rare genetic disorders *via* RNAseq. **(A)** Different machine learning and statistical approaches have been adapted or developed and applied to facilitate diagnosis *via* aberrant expression, aberrant splicing, and mono-allelic expression detection. For aberrant expression, the distribution of read counts is depicted, with outliers in red. For detection of aberrant splicing, an exon skipping event is depicted in red. For mono-allelic expression, a SNV with MAE of the alternative allele A is shown. Below each diagram the different methods used to detect RNAseq outliers of the corresponding cases are provided. **(B)** Different types of alternative splicing scenarios are depicted. Different mRNA isoforms can be generated from a single pre-mRNA through exon skipping, exon creation, exon truncation, exon elongation, and intron retention. Exons are represented as green and red boxes. Introns are illustrated as solid lines between the boxes. **(C)** A subset of a gene model is shown with split reads going from splice donor D to splice acceptors A_1_ (blue) and A_2_ (purple), and non-split reads spanning the exon–intron boundary at the splice donor D (yellow). These reads can then be converted into the percent spliced-in (*ψ*) and the splicing efficiency (*θ*) metrics using the formulae below the gene model.

Counts of reads aligned to genes are used as the basis for quantifying gene expression and detecting expression outliers (Figure 2A). These read counts should be normalized for sample sequencing depth by dividing the total depth or using size factors (Anders et al., 2013). Read counts can additionally be controlled for gene length resulting in the metrics reads per kilobase million (RPKM) (Mortazavi et al., 2008) or transcripts per million (TPM) (Wagner et al., 2012). Gene expression profiles are known to covariate due to both physiological regulation and technical artifacts. Therefore, several statistical methods, including principal component analysis (PCA) and surrogate variable analysis (SVA), have been adapted or specifically developed to correct for technical artifacts keeping biological-relevant signals. PCA has been used to cluster and reduce the dimension of gene expression matrices (Yeung and Ruzzo, 2001). Further, it has been shown that the top principal components can effectively explain the variation of gene expression (Ma and Dai, 2011; Todorov et al., 2018) and has been thus used to remove technical covariation. The SVA algorithm was introduced to capture gene expression heterogeneity thereby increasing biological accuracy and reproducibility of analyses in genome expression studies (Leek and Storey, 2007). Finally, using probabilistic estimation of expression residuals (PEER) on gene expression data outputs hidden factors explaining the expression variability (Stegle et al., 2012). All three methods are applied to remove unwanted covariation and improve gene expression data analysis.

The systematic implementation of gene expression analysis to detect potentially disease-associated genes causing aberrant expression in affected individuals has been successfully used as a complementary method in four studies by detecting expression outliers using distinct approaches (Kremer et al., 2017; Brechtmann et al., 2018; Frésard et al., 2019; Gonorazky et al., 2019). All approaches have been used in a gene-specific analysis, even if different isoforms of a gene are presented. One established way used to define an outlier data point is via its Z-score. Z-scores are defined as the difference between the observed value and the mean of the population, divided by the standard deviation of the population. Outliers are then commonly defined as observations with a |Z-score| greater than a cutoff, depending on the research field.

Gonorazky et al. (2019) applied this Z-score approach in a two-step procedure. First, genes with an RPKM |Z-score| ≥ 1.5 were defined as candidate outliers. Second, these candidates were compared to a control group and designated as outlier genes if their expression had at least a two-fold change with respect to the mean of the control group. Multiple testing was performed using Bonferroni’s method, yielding a median of 17 aberrantly expressed neuromuscular-related genes per sample. This allowed the detection of six causal genes out of 25 samples in a cohort of rare muscular disorders. While this approach led to some diagnoses, it lacks normalization for confounders and always requires the comparison with a control group from the same tissue as the affected samples. The Z-score approach has also been used in expression quantitative trait loci studies of common disorders (Smail et al., 2020).

**FIGURE 3:**
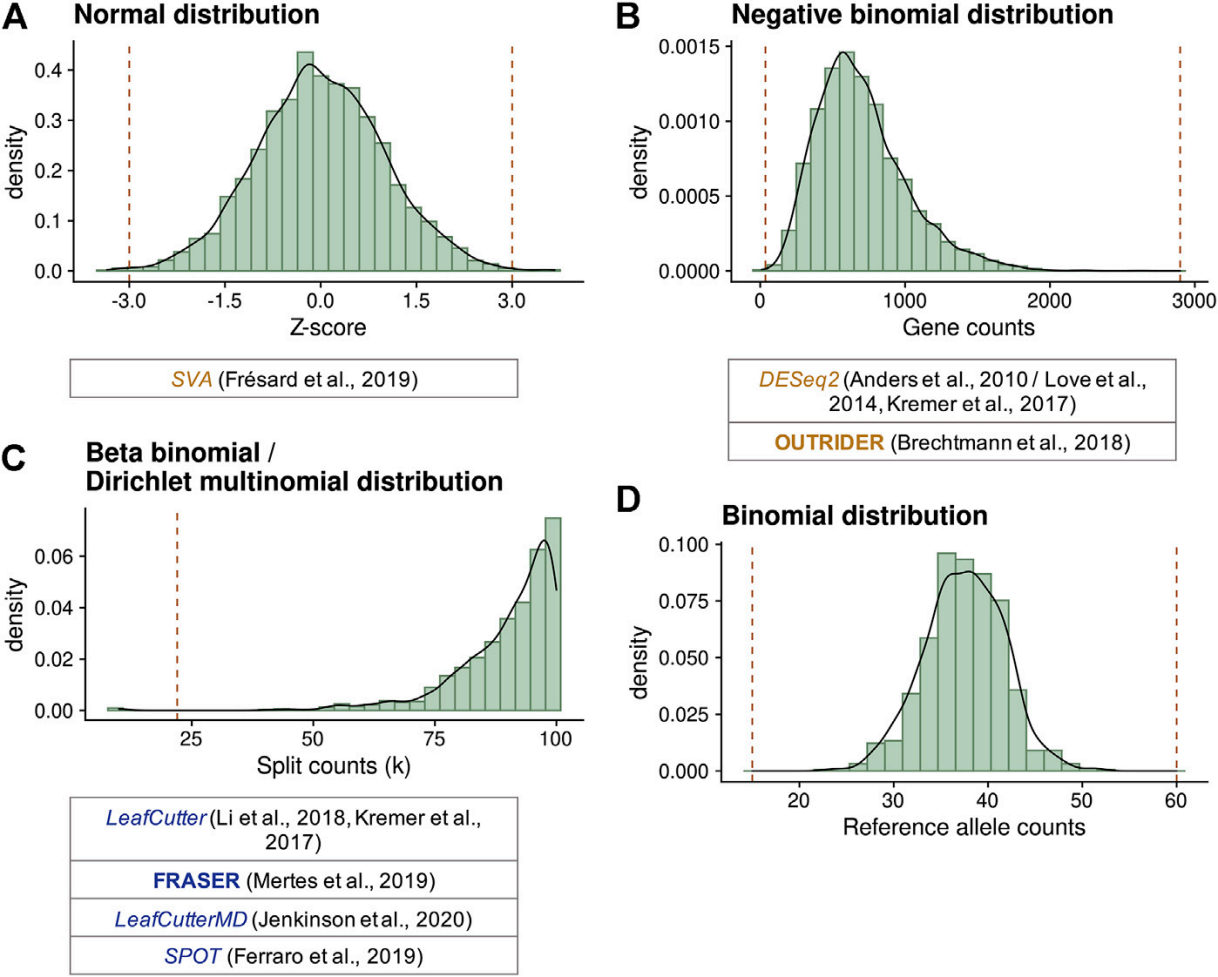
Statistical distributions used to model gene expression data and compute outliers. **(A)** Standard normal distribution simulating standardized residual or Z-scores. Here, cutoffs defining outliers at ±3 (vertical lines) were selected. **(B)** Negative binomial distribution simulating gene counts with a mean of 700 and dispersion of 5. Values lower than 36 or greater than 2,899 (vertical lines) have a probability lower than 10^−5^ to occur, reflecting expression outliers. **(C)** Beta-binomial distribution simulating the distribution of split counts (n (D, A)) given a total of 100, with a *ψ* expectation of 0.9 and a correlation of 0.1. Values lower than 22 (vertical line) have a probability lower than 10^−5^ to occur, reflecting splicing outliers. Dirichlet-multinomial distributions are multidimensional extensions of beta-binomial distributions. **(D)** Binomial distribution simulating the counts of each allele on a selected variant with a total of 75 counts and a probability of 0.5. Biological-relevant cutoffs at 20 and 80% of allelic ratio (15 and 60 counts) indicate MAE. Extensions of this distribution have been proposed to account for the genetic variation by ANEVA-DOT and the MAE test of Kremer et al. (2017). Below each diagram, different methods using these distributions are given.

Another way of defining outliers is by performing a statistical fit in the whole population and testing the distribution of the residuals (i.e., the difference between the real and the predicted value). This approach was used by Frésard et al. (2019) by applying a regression model on corrected TPMs using surrogate variables (SVs) and regression splines. In their cohort, the top two SVs significantly correlated with sequencing batch and sequencing facility. Adding the top two SVs and regression splines decreased the variation of the residuals, decreased the number of outlier genes per sample, and increased the coefficient of determination (*R* ^2^). Z-scores were generated from the residuals of this model, and values with a |Z-score| ≥ 2 were classified as outliers (Figure 3A), resulting in a median of 343 genes per sample. This very high number of outliers can be explained by the approach lacking multiple testing. Nevertheless, it allowed them to detect causal variants in four out of 80 samples in a cohort of various genetic disorders.

Kremer et al. (2017) adapted the method for differential expression DESeq2 (Love et al., 2014) and used it in a one vs. rest fashion. This approach models the counts using a negative binomial distribution parameterized with a mean and dispersion (Figure 3B). The mean was estimated taking into account size factors, batch, sex, and biopsy site of each sample, while the dispersion was specific for each gene. Gene-sample combinations with an adjusted *p*-value, using Hochberg’s method, lower than 0.05 and |Z-score| > 3 were classified as outliers, deeming a median of one outlier per sample. Four out of 48 cases were diagnosed using this approach. In this case, the correction for sample covariation was performed using known factors; therefore, latent confounders were not taken into account.

OUTRIDER was the first method applying a machine learning model to detect gene expression outliers (Brechtmann et al., 2018), instead of adaptations of methods used for the detection of differential expression. By using a denoising autoencoder, OUTRIDER controls for common covariation observed in gene expression. Autoencoders are machine learning models introduced to find low-dimensional representations of high-dimensional data (Hinton and Zemel, 1993). They achieve this by learning certain features from the data distribution by encoding it into a black-box representation and decoding it to denoised data (Hinton and Zemel, 1993). A subclass of autoencoders, called denoising autoencoders, is specialized to reconstruct corrupted high-dimensional data by exploiting correlations in the data (Vincent et al., 2008). OUTRIDER is taking advantage of this unsupervised learning method and applies it on gene-level counts (Brechtmann et al., 2018). Log-centered, size factor normalized gene counts are used as input for the autoencoder. The output is the parameters of the negative binomial distribution that the gene counts are assumed to follow, which are the expected counts and dispersions for each gene (Figure 3B). Multiple testing is performed on two-sided *p*-values using the Benjamini–Yekutieli false discovery rate (FDR) method, which holds under positive dependence because of gene co-expression. Expression outliers are defined as the gene-sample combinations with an FDR ≤0.05, resulting in a handful of outliers per sample, depending on the number of samples. In a subset of GEUVADI’s cohort (Lappalainen et al., 2013) comprising 100 control samples from different sequencing centers (CNAG CRG, *N* = 31; ICMB, *N* = 28; and UNIGE, *N* = 41) and ancestries (British, *N* = 12; Finnish, *N* = 26; Tuscan, *N* = 22; Utah, *N* = 16; and Yoruba, *N* = 24), OUTRIDER’s denoising autoencoder was able to control for covariation (Yépez et al., 2021b). The sample size of each subgroup was relatively similar; therefore, the autoencoder is yet to be tested in cases where a subgroup is substantially underrepresented. Murdock et al. (2021) applied OUTRIDER in a cohort of 78 DNA-unsolved patients with diverse disorders and diagnosed five of them with aberrantly expressed genes. OUTRIDER was also applied to a cohort of 303 rare disease patients sequenced in the same center but from different ancestries (mostly European and Asian), which led to the identification of 26 aberrantly expressed disease causal genes (Yépez et al., 2021a).

Currently, OUTRIDER is the only available automated method to compute expression outliers from gene expression matrices. It outperformed methods that use Z-scores on counts normalized using PEER and PCA in three different benchmarks. First, it achieved a higher precision in recovering injected outliers in simulations. Second, OUTRIDER had a higher enrichment of rare moderate and high impact variants among outliers, as shown in GTEx samples that there is a strong association between rare variants and expression outliers (Li et al., 2017). Third, it was able to recover all the five pathogenic events from the Kremer et al. (2017) dataset, while PCA missed two and PEER missed one of the five pathogenic events. Larger datasets with more confirmed pathogenic variants would further improve benchmarking.

After obtaining an expression outlier in a disease-associated gene, it is mandatory for a molecular diagnosis to identify causative rare genetic variants. Filters including allele frequency, computational predictions, segregation, and incurrence in unaffected controls are applied. The variants can be located in regulatory regions like enhancers, promoters, or suppressors of the corresponding gene, but also in the coding or intronic regions affecting splicing or creating a nonsense codon causing NMD. In Yépez et al. (2021a), using WES, the cause of aberrant expression remained elusive in 65% of outliers and without the identification of a causative variant, the case remains undiagnosed. This fraction can be further reduced by using WGS, allowing the discovery of structural variants, which explain up to 25% of expression outliers (Ferraro et al., 2020). A significant fraction of expression outliers can be explained by NMD affecting a single allele only, showing the sensitivity of RNAseq studies (Li et al., 2017; Ferraro et al., 2020; Yépez et al., 2021a). In the majority of cases solved *via* aberrant expression, the causal variant affects splicing. Some of the variants have been prioritized after WES; however, they usually remain as variants of uncertain significance. A large fraction of variants are outside the splice region and frequently deep intronic. Without detection of aberrant splicing, they would not have been prioritized. In addition, in some cases, aberrant expression was caused by deletions in the 5’UTR (e.g., NM_004544.3 c.-99_-75del causing 50% depletion in *NDUFA10*) or promoter regions (e.g., NM_016617.2 c.-273_-271del causing 40% depletion in *UFM1*) (Yépez et al., 2021a).

## Splicing Outliers

The concept of alternative splicing was initially introduced in 1978 based on the discrepancy between human protein-coding genes (∼25,000) and human proteins (>90,000) (Gilbert, 1978). More than 95% of human genes undergo alternative splicing, acting to a certain extent in a tissue, or development-specific, or signal transduction–dependent manner (Pan et al., 2008; Nilsen and Graveley, 2010). Alternative splicing gives rise to different isoforms of the mature mRNA of a gene (Baralle and Giudice, 2017). Various forms of alternative splicing are known, including exon skipping, generation of new exons, exon truncation, exon elongation, and intron retention (Figure 2B). Alternative splicing is strictly regulated, and aberrant splicing is an underlying cause of genetic diseases (Wang et al., 2015). The splicing mechanism is complex and variable. Even without genetic variation, it is difficult to quantitatively predict splicing. Functional consequences of rare variants within splice regions are challenging to predict, especially in the case of deep intronic variants. Moreover, splice defects are quantitative, often resulting in multiple isoforms with different frequencies. Analysis of RNAseq using patients’ samples enables the evaluation of splice variant consequences and detection of aberrant splice events, *de novo* and not predicted by DNA sequence.

To quantify splicing, reads spanning from the donor site of an exon to the acceptor site of another exon (split reads, n (D, A)) and reads overlapping an exon–intron boundary (non-split reads, n (D) for donor and n (A) for acceptor site) are counted and aggregated per junction (Figure 2C). These can then be converted into the intron-centric metrics percent-spliced-in (*ψ*) and splicing efficiency (*θ*) (Pervouchine et al., 2013). The *ψ* index is computed as the ratio between reads mapping to the given intron (n (D, A)) and all split reads sharing the same donor (*ψ*_5_) or acceptor site (*ψ*_3_), respectively. For the detection of partial or full intron retention, the splicing efficiency metric, defined as the ratio of all split reads and the full read coverage at a given splice site, is used (Figure 2C).

To detect aberrant splicing in patient samples suffering from rare genetic disorders, distinct, already available methodologies were applied to RNAseq data. One approach, used by Cummings et al. (2017) and Gonorazky et al. (2019), to detect aberrant splicing consists of comparing normalized split reads of affected individuals against those of controls and other affected samples. In both studies, normalized split reads were obtained by dividing them with the maximum number of split reads of a shared exon–intron junction. In Cummings et al. (2017), aberrant junctions were those whose normalized value was the highest in the sample of interest and twice or higher than the next highest. This approach resulted in a median of 190 aberrantly spliced genes per sample and allowed them to diagnose 10 out of 50 individuals with muscular disorders. In Gonorazky et al. (2019) for a junction to be aberrant, either the donor or the acceptor site cannot be annotated in GENCODE, must not be present more than five times in control samples (from GTEx), and it has to be unique among the affected cohort. On median, five aberrant junctions per sample were identified and led to the diagnosis of eight out of 25 samples with neuromuscular disorders. In a second approach for the detection of aberrant splicing, Kremer et al. (2017) adapted the method LeafCutter (Li et al., 2018), originally developed to test for differential usage in intron clusters between two groups, to work in a one vs. rest manner. LeafCutter’s Dirichlet-multinomial’s approach returns a *p*-value per intron per sample, later corrected for multiple testing using Hochberg’s method. Outliers were defined as those with an adjusted *p*-value < 0.05, yielding a median of five outliers per sample. Although the method contributed to the diagnosis of three out of 48 cases, it does not control for sample covariation. However, Frésard et al. (2019) showed the existence of sample covariation on *ψ* values. Therefore, they applied a similar approach as they did for the aberrant expression analysis but regressed out principal components accounting for 95% of the variation instead of SVs and splines. The first three principal components are correlated with RIN number, batch, and sequencing facility. Splicing outliers were defined as those with a |Z-score| ≥2, yielding on average 540 outliers per sample. This approach allowed them to diagnose two out of 80 patients. Frésard’s methodology nevertheless is limited by not offering control for multiple testing and low power to detect aberrant splicing in splice sites with low reads.

Bearing in mind the limitations of the approaches described, three specialized methods to systematically detect aberrant splicing were developed: FRASER, LeafCutterMD, and SPOT. FRASER (Find Rare Splicing Events in RNAseq) is an approach combining machine learning and statistical models to detect aberrant splicing from RNAseq data (Mertes et al., 2021). Using the same rationale as OUTRIDER for detecting aberrant expression, FRASER uses a denoising autoencoder automatically controlling for latent confounders. Further, FRASER fits for each intron a beta-binomial distribution on the intron-centric metrics *ψ*_5_, *ψ*_3_, and *θ*, independently. The distribution is parameterized with a sample intron-specific proportion expectation and an intron-specific correlation (Figure 3C). *p*-values are computed and two multiple testing steps are performed, the first at the junction level using Holm’s method (Holm, 1979), and the second at the gene level using Benjamini–Yekutieli’s method (Benjamini and Yekutieli, 2001). Splicing outliers are defined as the intron-sample combinations with an FDR <0.10. A |*Δ ψ*| > 0.3 is recommended as an additional filter for the identification of pathological-relevant variation, where it corresponds to the difference between the observed *ψ* and the expected *ψ*. Application of FRASER in the rare disease cohort from Kremer et al. (2017) identified all three previously detected pathogenic splicing aberrations, plus an intron-retention event missed by LeafCutter, and a synonymous variant causing a splice defect missed by Kremer et al. (Mertes et al., 2021). FRASER was also applied to the GEUVADIS multicenter and multi-ancestry cohort and was able to remove sample covariation for all metrics (Yépez et al., 2021b). In addition, FRASER has been used by Murdock et al. (2021) leading to the diagnosis of four out of 78 subjects and by Yépez et al. (2021a) leading to the diagnosis of 19 (12 in combination with aberrant expression) subjects from various ancestries.

LeafCutter for Mendelian disease (LeafCutterMD) was introduced in 2020 as an adaptation of LeafCutter (Li et al., 2018) to detect outlier splicing events (Jenkinson et al., 2020). Like its predecessor, LeafCutterMD uses an intron-based clustering approach, in which all split counts belonging to the same cluster are modeled together. Thus, it uses a Dirichlet-multinomial distribution, which is a generalized higher-dimensional version of the beta-binomial distribution (Figure 3C). Both distribution parameters allow it to account for biological variability and uncertainties due to statistical sampling. In simulations, the power of LeafCutterMD (1—the probability of a Type II error) is up to 50% higher than the power of LeafCutter (Jenkinson et al., 2020). Two-sided *p*-values are estimated for each intron of each cluster, later being corrected for multiple testing. In the same study, LeafCutterMD was applied to a cohort of 128 individuals with an undiagnosed genetic disease, out of which three were found to have splicing aberrations by manual inspection after variants were detected on them. LeafCutter failed to identify all three of them as outliers, while LeafCutterMD did identify them, demonstrating its improvement and application to rare disease cohorts. However, it does not account for latent confounders.

SPOT (Splicing Outlier deTection), the third novel approach developed to detect aberrant splicing, fits a Dirichlet-multinomial distribution on each of the intron clusters generated by LeafCutter (Ferraro et al., 2020), thus obtaining estimates of the distribution parameters for each cluster. Using these parameters, it generates 1 million random values and computes the Mahalanobis distance (Mahalanobis, 1930) of each of these values to the Dirichlet-multinomial distribution. This distance takes into account the covariance of the split reads. Empirical *p*-values are computed for each sample-intron cluster by comparing its Mahalanobis distance against the 1 million simulated ones. This method is yet to be tested in a rare disease cohort.

By inserting outliers in samples from skin and brain from GTEx, FRASER obtained higher precision in detecting the simulated outliers at all recall levels than SPOT and LeafCutterMD (Mertes et al., 2019). Following the principle that rare variants in the splice regions can disrupt splicing (Rivas et al., 2015), Mertes et al. (2021) also benchmarked by performing an enrichment of rare variants in the splice region among splicing outliers called by the three specialized methods. FRASER obtained a higher enrichment of rare splice-region variants and variants predicted to affect splicing [using MMsplice (Cheng et al., 2019)] than SPOT and LeafCutterMD across all tissues from the GTEx dataset (GTEx Consortium, 2017).

The variability of splicing is much higher than of gene expression and can often not be linked to a genetic variant in cis (Ferraro et al., 2020; Yépez et al., 2021a). Published pathogenic splice-disrupting variants discovered *via* RNAseq are usually within the annotated splice region and, as such, likely to cause aberrant splicing, or within the coding region or deep intronic regions activating novel splice sites. Only a minor fraction of these variants was functionally validated. Methods like MMSplice (Cheng et al., 2019) or SpliceAI (Jaganathan et al., 2019) can be further used to pinpoint which variant is most likely causing the splicing aberration.

## Mono-Allelic Expression

Besides aberrant expression and splicing, RNAseq contains information about allelic expression, the expression of the maternal and paternal haplotype of an individual. In the case of allele-specific expression (mono-allelic expression, MAE) one allele is silenced and only the other allele is expressed. MAE is an extreme form of allelic imbalance. The reasons for MAE can be diverse and may be driven by loss-of-function genetic variants or epigenetic effects, such as imprinting of autosomal genes (Santoni et al., 2017) or inactivation of the X chromosome (Knight, 2004; Tukiainen et al., 2017). Assuming a recessive mode of inheritance, heterozygous variants are not considered to be disease causing if present alone (Albert and Kruglyak, 2015). The analysis of MAE can prioritize such rare heterozygous variants identified by WES (Kremer et al., 2017).

Detection of mono-allelically expressed genes relies on counting the expressed alleles at genomic positions of single-nucleotide heterozygous variants (SNVs, Figure 2A). Thereafter, it is tested whether these counts are evenly distributed among both alleles, or whether there is a pronounced skew toward one of the alleles (Figure 3D). These counts can be transformed into ratios by dividing the counts aligning to each allele over the total counts. Five methods have been developed to detect MAE in the context of rare genetic disorders.

Cummings et al. (2017) computed a 95% confidence interval of the mean allele balance for each gene using GTEx samples and compared the balance of the affected samples against it. Frésard et al. (2019) scaled reference allele ratios across samples thus converting them into Z-scores, ranked them, and further used them to support the findings from aberrant expression. Gonorazky et al. (2019) scaled alternative allele ratios into Z-scores and compared them against the values from GTEx samples which directed to the cause of disease in three cases.

Kremer et al. (2017) proposed a negative binomial test with a fixed dispersion for all genes. The negative binomial distribution accounts for a dispersion parameter not present in the binomial distribution. The test outputs a *p*-value for each SNV-sample combination which is later corrected for multiple testing. To distinguish between allelic imbalance and MAE, alleles with an alternative allele ratio greater than 0.8 or lower than 0.2 and Benjamini–Hochberg adjusted *p*-values < 0.05 were considered to be mono-allelically expressed.

Mohammadi et al. (2019) introduced ANEVA, a generative model to quantify genetic variation (*V* ^*G*^) in gene dosage (i.e., expression) within a population. Using *V* ^*G*^, they further developed ANEVA-DOT which implements a binomial-logit-normal test on each SNV to detect MAE. The rationale is that the variance of each gene is different and should be taken into account when testing for MAE. Moreover, it takes into account reference allele alignment bias and the probability of the allelic count to be incorrect (i.e., assignment to the reference allele when it corresponds to the alternative or vice versa). MAE variants are those with an FDR <0.05. ANEVA-DOT was applied to the cohort of 70 rare Mendelian muscle dystrophy and myopathy patients described in Cummings et al. (2017). In that cohort, 16 out of 70 patients had MAE pathogenic variants. ANEVA-DOT was able to recover all of them, plus it outperformed other tests that use binomial and beta-binomial distributions by obtaining the highest recall of the 16 true causal genes and the lowest number of reported outlier genes. Moreover, ANEVA-DOT detected a novel MAE in one proband from this cohort leading to a new diagnosis (Mohammadi et al., 2019). In order to estimate *V* ^*G*^, a large cohort with DNA and RNA sequence information is needed. Even using the large GTEx data collection (with a median of more than 200 samples per tissue), ANEVA estimates for *V* ^*G*^ could only be computed for 4,962 genes (in median per tissue).

No formal benchmark has been done between the negative binomial method and ANEVA-DOT, and the application of ANEVA-DOT is currently limited by not providing genome-wide calling. Unlike for aberrant expression or splicing, one limitation of MAE is that it requires a detected variant to be able to compute the allelic counts. Obtaining MAE in genes constrained to variation, shorter genes, or ethnicities similar to the reference genome is, therefore, limited. So far, the added value of MAE has been lower with respect to that of aberrant expression or splicing and often in combination with aberrant expression (Kremer et al., 2017; Gonorazky et al., 2019; Yépez et al., 2021b).

## Limitations and Future Perspectives of RNAseq in the Molecular Diagnosis of Rare Disorders

The application of technologically and bioinformatically advanced next-generation sequencing methodologies has increased the number of patients with rare genetic disorders getting a clear molecular diagnosis. Nevertheless, a large fraction of those patients remains unsolved by DNA sequencing. Integration of RNAseq approaches appears promising to improve the diagnostic yield, yet several challenges remain to be addressed in the future.

Gene fusion, which consists of genetic material from different genes being merged and transcribed together, can be detected from RNAseq data and has proven to be successful in cancer diagnostics (Mertens et al., 2015; Dai et al., 2018). A variety of methods including Manta (Chen et al., 2016), ChimeraScan (Iyer et al., 2011), or STAR-Fusion (Haas et al., 2019) have been developed to call gene fusions. Statistical improvements, such as the inclusion of factors like allele frequency or repetitive matching to better distinguish between “bona fide gene fusions” from artifacts in CICERO will further enhance the quality of the fusion calls (Tian et al., 2020). Early studies have shown that calling gene fusions can also lead to diagnose rare diseases (Oliver et al., 2019), but its systematic application is yet to be explored.

Although all patients’ cells share an almost identical genome, each cell type and subtype displays different levels of gene expression and gene isoforms. Moreover, it is assumed that not only different cell types exhibit various transcriptomes but also that the transcriptome of cells within a tissue varies (Shalek et al., 2014). Given the tissue-specific expression of genes and mRNA isoforms, analysis of disease-relevant tissues is for many diseases of great value for the interpretation of genetic variants (Wang et al., 2008; Melé et al., 2015; Cummings et al., 2017). However, obtaining biopsies of disease-relevant tissue is unfeasible for many rare genetic diseases. Blood, for instance, is the most easily accessible tissue, yet the blood transcriptome is not well suited for the analysis of a number of rare diseases (The GTEx Consortium, 2015; Cummings et al., 2017; Gonorazky et al., 2019). Fibroblast or myoblast cell lines based on skin or muscle biopsies are often used as surrogates. Patient cell lines are usually only available to a limited extent, and indefinite proliferation is not always possible. Consequently, transformed, immortalized cell lines, or animal models have been applied as disease models. Yet, neither model provides the possibility to fully replicate the physiology of patient cell lines nor do they consider aspects of distinct gene expression and splicing in various subgroups of tissues (Anderson and Francis, 2018). By reprogramming mature patient cell lines into patient-specific induced pluripotent stem cells (iPSCs), the patient’s genotype is retained. Patient-specific iPSCs, where as many as 27,046 protein-coding and nonprotein-coding genes are expressed, provide a suitable model to study the consequences of the disease genotype on RNA level (Hamazaki et al., 2017; Bonder et al., 2019). Due to the differentiation and self-renewal properties of iPSCs, their differentiation into disease-relevant tissues is an approach to overcome the limited accessibility of tissues. Generation and differentiation of iPSCs carrying disease-relevant mutations has already shown the ability to replicate the patients’ phenotype. Simulating rare genetic diseases with the help of patient-specific iPSCs now further offers the possibility to analyze the transcriptome of the tissue of interest and thus to investigate new pathomechanisms (Sterneckert et al., 2014).

Some genetic variants affect the expression of a gene in a cell type–specific manner. Transcriptome analysis of an entire cell population, the so-called bulk RNAseq, is typically based on RNA extracted from tissues that may contain several cell types. These bulk RNAseq analyses provide a bulk average and are not cell type–specific. When analyzing a blood sample, the sensitivity for the detection of outliers depends on the contribution of the affected cell types in the blood sample, and the composition of different cell types may change over time. Whole transcriptome sequencing of single cells (scRNAseq) has the potential to identify and characterize rare cell populations (Hwang et al., 2018; Lederer and La Manno, 2020). ScRNAseq has generated comprehensive compendiums of cell types per tissue and identified cell types that play a critical role in genetic diseases, which can guide cell type–specific investigations (The Tabula Muris Consortium et al., 2018; Aizarani et al., 2019). In particular, scRNAseq has already been used as a powerful tool in fields such as immunology (Mizoguchi et al., 2018; Zhang, 2019), cancer (Cheng et al., 2021; Velten et al., 2021), and neurological diseases (Jäkel et al., 2019; Mathys et al., 2019). ScRNAseq of diseased tissues or patient-specific iPSCs could further advance the precision medicine approach in the field of rare genetic disorders (Choi et al., 2020).

Within the process of alternative splicing of human premessenger RNAs, multiple mRNA isoforms are generated from a single gene leading to differences in protein isoforms, structure, and function (Park et al., 2018). mRNA isoform-specific defects can result in different diseases. Standard and commonly used RNAseq platforms generate short reads of 150–300 bases, generally spanning at most two exon junctions per read. Short read RNAseq methodologies are constrained by their need for computational reconstruction of single short reads into entire transcripts (Steijger et al., 2013). Most of the human mRNAs, however, are longer than 3 kb (Piovesan et al., 2019) and with more than 100 kb Titin represents the longest disease-relevant human transcript (Bang et al., 2001). Hence, short read sequencing methods are not the best method of choice for detection and quantification of mRNA isoform expression and do not phase alternative exons. Recently developed long read sequencing technologies, producing reads of up to 100 kb, enable the accurate identification and quantification of mRNA transcript isoforms (Oikonomopoulos et al., 2020; Uapinyoying et al., 2020). Various bulk or single-cell long read RNAseq approaches revealed a significant number of missing mRNA isoforms in the current transcript annotations, suggesting a more complex mRNA isoform scene than previously assumed (Sharon et al., 2013; Tilgner et al., 2013; Anvar et al., 2018; Gupta et al., 2018; Glinos et al., 2021). Moreover, long reads improve sequence alignment, reduce the number of multi-mapped reads, allow phasing of variants, and increase the number of split-reads thereby improving the calling of aberrant splicing (Mantere et al., 2019; Amarasinghe et al., 2020; Mitsuhashi and Matsumoto, 2020). The power of long read transcriptome data in disease diagnostics has already been successfully demonstrated in diseases, such as Alzheimer’s disease (De Roeck et al., 2017) or X-linked dystonia parkinsonism (Aneichyk et al., 2018). Methods to model and normalize gene expression might need to adapt as quantification of longer reads might statistically differ from that of short reads.

Transcriptome sequencing is used to assess the effects of variants on gene expression. Thresholds for pathological gene expression are not yet established, and aberrant RNA expression could be compensated on the protein level and not necessarily cause disease. Buffering mechanisms exist and transcript levels cannot be directly translated to protein levels (Battle et al., 2015). Moreover, low RNA expression levels not called as a statistical outlier may be pathologically relevant. Therefore, for the clinical interpretation according to the ACMG/AMP criteria, additional functional studies, such as proteomics and functional assays, are often needed for the validation of aberrant RNA expression (Richards et al., 2015). With an increasing number of patients with aberrant expression, thresholds of aberrant gene expression can be established. Proteomic approaches have advanced, now allowing protein quantification, study of protein–protein interactions, and identification of posttranslational modifications by high-throughput proteomics (Wang et al., 2014; Stenton and Prokisch, 2020). For example, integrated genome, transcriptome, and proteome analyses allowed the validation of a rare variant causing aberrant gene expression of genes such as TIMMDC1 (Kremer et al., 2017), *PTCD3* (Borna et al., 2019), or *MRPS34* (Lake et al., 2018). More recently, Kopajtich et al. (2021) demonstrated the effectiveness of integrating genomics, transcriptomics, and proteomics in a systematic diagnostic application to discover the genetic cause of 20% of unsolved patients with suspected mitochondrial disorders. These recent advancements in bioinformatic approaches help to combine these multi-omics data and enable their holistic analysis.

Due to the expanding availability and automation of DNA and RNA sequencing, they are increasingly applied in diagnostics. While the demand is decreasing on the wet lab side, the increasing throughput of such methods and generation of large data sets requires larger computational infrastructure, adaptation, and automation of bioinformatic algorithms. Depending on the algorithms used, memory consumption and computation time may increase exponentially, logarithmically, or otherwise depending on the size of the dataset to be analyzed (Baichoo and Ouzounis, 2017). The machine learning approaches OUTRIDER and FRASER led to a shorter computational time with respect to DESeq and LeafCutter and therefore speed up analysis. Moreover, both tools have been integrated in a computational workflow, DROP, which also automates the preprocessing and quality control steps (Yépez et al., 2021b). Besides seeking to obtain high precision, newly developed methods must also pursue yielding results in a low computational time and being able to model hundreds or thousands of samples together.

Another challenge remaining in the analysis of RNAseq data is the replication of outliers. There are no studies about replication of outliers using multiple biopsies from the same tissue from the same patient. However, Ferraro et al. (2020) and Mertes et al. (2021) performed replication analyses using different tissues from the same individuals from the GTEx cohort. Ferraro et al. (2020) found that a median of 5.1% expression outliers, 8.7% of splicing outliers (using SPOT), and 10.7% of mono-allelically expressed genes (using ANEVA-DOT) that were detected as aberrant in one tissue were replicated in another. This was confirmed by Mertes et al. (2021) who found that more than 80% of splicing outliers are found in only one tissue, for SPOT, LeafCutterMD, and FRASER. These low replication numbers likely reflect the differences between the tissues but could also indicate variation within cells of the same tissue. Pooled CRISPR screens combined with scRNAseq have emerged as powerful tools for profiling the functional effects of genetic variants at the single-cell level (Adamson et al., 2016; Dixit et al., 2016; Jaitin et al., 2016; Datlinger et al., 2017). Using different cell types in parallel, those approaches have the potential to study the tissue specificity of splice variants and replication across tissues.

To properly normalize and model read counts and split reads, a minimum number of samples are needed. OUTRIDER and FRASER recommend at least 60 and 30 samples, respectively. Integrating affected with external control samples can help reach this minimum, as long as they were originated from the same tissue and sequenced using a similar protocol (Frésard et al., 2019; Yépez et al., 2021b). When integrating samples, it is recommended to first inspect the plots of the normalized counts (e.g., *via* heatmaps or principal components), especially if the affected samples are too diverse with respect to the controls (e.g., ancestry, age, or disease). Gene expression could depend on developmental status. Therefore, it is recommended to consider adequate control samples. For example, for pediatric cases, the CZI pediatric cell atlas or developmental GTEx can serve as appropriate control datasets (Taylor et al., 2019). Gene coverage also plays a role in outlier detection. Yépez et al. (2021b) showed that less expression and splicing outliers were detected in samples with lower sequencing depth (∼30 vs. ∼85 million reads). A systematic study to find the minimum coverage a gene and a junction should have in order to be detected as an outlier, and whether genes that are very highly expressed tend to be more prone to be called as expression or splicing outliers, is pending. Likewise, the sequencing protocol might influence the detected outliers. The total RNAseq protocol contains more immature splice transcripts in comparison to poly-A enriched. Those immature splice transcripts increase the noise and can be misinterpreted as aberrant splicing. On the other hand, using a poly-A enriched protocol might have an impact on detecting aberrant splicing on the 5’ end of larger genes. In a poly-A enriched cohort, Yépez et al. (2021a) showed that genes with many exons tend to have more splicing outliers than genes with fewer exons, even after correcting for multiple testing inside each gene, but there was no information about the position of the aberrant junctions inside each gene. An analysis of a dataset with samples from the same donors sequenced using both protocols [such as the one in Chen et al. (2020)] will be valuable.

Finally, the approaches introduced here used different thresholds and cutoffs to define outliers and to filter out genes or junctions with low expression. Fine-tuning thresholds and cutoffs to obtain the desired precision-recall balance is of utmost importance and need to be adopted to the question. Thresholds can be defined by biological and statistical significance. A reduction to less than half of gene expression with respect to the median could reflect a pathological situation specifically in genes prioritized by the finding of rare DNA variants. If the data are explored to discover aberrant expression, considering the high number of tests (in the 10,000 s for gene expression, 100,000 s for aberrant splicing, and in between for MAE), multiple testing correction is necessary. These tests are not independent due to gene co-expression, split counts on a same cluster being coupled, and allelic expression being the same among all SNVs of a gene. Many multiple testing methods have been implemented, yet no agreement on a common guideline has been reached.

## Conclusion

In diagnostics of rare disorders, it is important to be able to evaluate the functional consequences of genetic variants. A correct molecular diagnosis enables to study the natural history and pathomechanisms of the disease which may lead to targeted therapy. The current diagnostic gap in DNA sequencing can in most cases be traced back to difficulties in variant prioritization. RNAseq has now been shown to be effective in increasing the diagnostic yield. Numerous diagnostic laboratories have already implemented DNA sequencing technologies and the establishment of RNAseq protocols in these laboratories would be easy and straightforward. Machine learning approaches have automated normalization and denoising of confounded RNAseq data, providing gene expression data in a high-throughput manner ready for analysis. Statistical methods have been adopted to the analysis of aberrant expression on the quantitative and qualitative level. However, pathological variants and statistical significance may need different thresholds. Statistical models optimized to control for false positive hits may be too stringent and miss pathological events. The limited number of positive controls for pathological aberrant expression results in an unsecure validation of established methods for diagnostics. With increasing datasets, this shortcoming has to be addressed in the near future and will force the establishment of guidelines. The application of machine learning is only in its beginning, and we foresee that deep learning methods will further improve the diagnostics of rare disorders.

## Abbreviations

ACMG/AMP, American College of Medical Genetics and Genomics and the Association for Molecular Pathology; ANEVA-DOT, analysis of expression variation–dosage outlier test; CADD, Combined Annotation Dependent Depletion; DNA, deoxyribonucleic acid; FDR, false discovery rate; FRASER, Find Rare Splicing Events in RNAseq; GTEx, Genotype-Tissue Expression; iPSCs, induced pluripotent stem cells; LeafCutterMD, LeafCutter for Mendelian disease; LIMS, laboratory information management system; MAE, mono-allelic expression; MMsplice, modular modeling of splicing; NMD, nonsense-mediated decay; OUTRIDER, OUTlier in RNAseq fInDER; PCA, principal component analysis; PEER, probabilistic estimation of expression residuals; PTM, posttranslational modification; RNA, ribonucleic acid; RNAseq, RNA sequencing; RPKM, reads per kilobase million; scRNAseq, single-cell RNA sequencing; SIFT, sorting intolerant from tolerant; SNV, single nucleotide variant; SPOT, SPlicing Outlier deTection; SVA, surrogate variable analysis; SV, surrogate variable; TPM, transcripts per million; *V* ^*G*^, genetic variation; WES, whole exome sequencing; WGS, whole genome sequencing.
